# Use of Fitness and Nutrition Apps: Associations With Body Mass Index, Snacking, and Drinking Habits in Adolescents

**DOI:** 10.2196/mhealth.6005

**Published:** 2017-04-25

**Authors:** Nathalie De Cock, Jolien Vangeel, Carl Lachat, Kathleen Beullens, Leentje Vervoort, Lien Goossens, Lea Maes, Benedicte Deforche, Stefaan De Henauw, Caroline Braet, Steven Eggermont, Patrick Kolsteren, John Van Camp, Wendy Van Lippevelde

**Affiliations:** ^1^ Food Chemistry and Human Nutrition Department of Food safety and Food quality University of Ghent Gent Belgium; ^2^ School for Mass Communication Research Faculty of Social Sciences KU Leuven Leuven Belgium; ^3^ Clinical Developmental Psychology Department of Developmental, Personality and Social psychology University of Ghent Gent Belgium; ^4^ Health Promotion and Education Departement of Public Health University of Ghent Gent Belgium; ^5^ Physical activity, Nutrition and Health Faculty of Physical Education and Physical Therapy Vrije Universiteit Brussel Brussel Belgium; ^6^ Nutrition and Food Safety Departement of Public Health University of Ghent Gent Belgium

**Keywords:** mhealth, adolescents, snacks, beverages, body mass index

## Abstract

**Background:**

Efforts to improve snacking and drinking habits are needed to promote a healthy body mass index (BMI) in adolescents. Although commercial fitness and nutrition mobile phone apps are widely used, little is known regarding their potential to improve health behaviors, especially in adolescents. In addition, evidence on the mechanisms through which such fitness and nutrition apps influence behavior is lacking.

**Objectives:**

This study assessed whether the use of commercial fitness or nutrition apps was associated with a lower BMI and healthier snacking and drinking habits in adolescents. Additionally, it explored if perceived behavioral control to eat healthy; attitudes to eat healthy for the good taste of healthy foods, for overall health or for appearance; social norm on healthy eating and social support to eat healthy mediated the associations between the frequency of use of fitness or nutrition apps and BMI, the healthy snack, and beverage ratio.

**Methods:**

Cross-sectional self-reported data on snack and beverage consumption, healthy eating determinants, and fitness and nutrition app use of adolescents (N=889; mean age 14.7 years, SD 0.8; 54.8% [481/878] boys; 18.1% [145/803] overweight) were collected in a representative sample of 20 schools in Flanders, Belgium. Height and weight were measured by the researchers. The healthy snack ratio and the healthy beverage ratio were calculated as follows: gram healthy snacks or beverages/(gram healthy snacks or beverages+gram unhealthy snacks or beverages)×100. Multilevel regression and structural equation modeling were used to analyze the proposed associations and to explore multiple mediation.

**Results:**

A total of 27.6% (245/889) of the adolescents used fitness, nutrition apps or both. Frequency of using nutrition apps was positively associated with a higher healthy beverage ratio (*b*=2.96 [1.11], *P*=.008) and a higher body mass index z-scores (zBMI; *b*=0.13 [0.05], *P*=.008. A significant interaction was found between the frequency of using nutrition and for the zBMI (*b*=−0.03 [0.02], *P*=.04) and the healthy snack ratio (*b*=−0.84 [0.37], *P*=.03). Attitude to eat healthy for appearance mediated both the fitness app use frequency-zBMI (*a* × *b*=0.02 [0.01], *P*=.02) and the nutrition app use frequency-zBMI (*a* × *b*=0.04 [0.01], *P*=.001) associations. No mediation was observed for the associations between the frequency of use of fitness or nutrition apps and the healthy snack or beverage ratio.

**Conclusions:**

Commercial fitness and nutrition apps show some association with healthier eating behaviors and BMI in adolescents. However, effective behavior change techniques should be included to affect key determinants of healthy eating.

## Introduction

In Flanders, Belgium, 18% of the adolescents between 14 and 16 years are overweight [1]. An unhealthy lifestyle characterized by physical inactivity [2], sedentary behavior [3], and unhealthy eating habits [4], plays an important role in the development of obesity. Typical unhealthy eating habits during adolescence are a low consumption of fruit, vegetables, and dairy products and an overconsumption of energy-dense snacks and sugar- and fat-rich beverages [5-9]. Health promotion programs to improve snack and beverage intakes are needed to promote a healthy body mass index (BMI) in adolescents.

Mobile phone use has significantly increased over the last decades, especially among adolescents and children [10-12]. With this increase, health-related apps have become widely spread [10,13-15]. Currently, 79,298 apps are available in the health and fitness category of iTunes and 75,058 in Google Play [16,17], the two leading app stores in Europe [14]. These apps are usually available in English or Dutch [14]. Among these apps, fitness and nutrition apps are the most popular and are typically used to improve fitness or eating habits [10,13]. Fitness and nutrition apps allow users to monitor their physical activity or food intake, provide information on the nutritional content of specific food items, and give instructions or demo videos for physical exercises [13,18,19]. Adolescents are highly skilled in using mobile phones and apps [20,21]. In 2014, 86% of the adolescents in Flanders owned a mobile phone and on average had 10-20 apps installed on the device [22]. Fitness and nutrition apps may thus be a promising, engaging, and affordable way to promote healthy lifestyle behaviors in adolescents [11,12,23].

Despite their potential for health promotion, little information exists about the use and effectiveness of commercially available fitness and nutrition apps [12,24]. Only a handful of studies, mostly in adults, have investigated the use of such apps and/or their relation with health [10,13,18,25,26]. The use of commercially available fitness apps was found to be associated with higher exercise levels, lower BMI, weight loss, and healthier eating [18,25], whereas the use of nutrition apps has been associated with better diet monitoring and weight loss [27,28]. Among adolescents, no differences in physical fitness were observed between fitness app users and nonusers during a randomized controlled trial [20]. To the best of our knowledge, no studies have investigated the use of existing fitness and nutrition apps and their relation with healthy eating habits and/or a lower BMI in adolescents.

Most existing fitness and nutrition apps for children, adolescents, and adults only contain a few effective behavior change techniques and might therefore have a limited capacity to facilitate behavior change [12,23,24,29]. Nevertheless, these apps are popular and perceived as useful and effective by the users [26]. Better evidence gathered via population-based studies on the usage patterns of such apps, their perceived utilities and benefits, and associations with health behaviors and BMI is needed [10,13,26,30].

To more fully comprehend the possible effects of nutrition and fitness apps on health behaviors, the mechanisms through which such apps influence behavior should also be explored [12,18,29]. Fitness and nutrition apps usually do not alter behavior directly, but they contain specific features that focus on key behavioral determinants [18,19,25,31]. Important intermediate determinants of healthy eating habits in adolescents are attitude to eat healthy, behavioral control, social norm, and social support [9,32-34]. Adolescents’ attitude to eat healthy is mainly determined by taste, appearance, and health concerns [35-37]. An assessment of intermediate determinants in adults found that the use of fitness apps was associated with a lower BMI through a higher self-efficacy to exercise and higher levels of exercise [18]. To the best of our knowledge, no study has investigated if commercially available fitness and nutrition apps target and/or positively influence key intermediate determinants of adolescents’ eating behaviors and anthropometrics.

This study first aimed to examine the use of fitness and/or nutrition apps and the associations between fitness and/or nutrition app use frequency and BMI, healthy snacking, and drinking habits (healthy snack or beverage ratio) in adolescents. It was expected that app use frequency would be related to a lower BMI and a higher healthy snack and/or beverage ratio. The combined influence of fitness and nutrition apps was also considered, as adolescents might use both fitness and nutrition albeit not always to the same extend [10,13]. Second, this study aimed to explore if the key behavioral determinants to eat healthy mediated the associations between fitness and/or nutrition app use and BMI, the healthy snack or beverage ratio. Perceived behavioral control to eat healthy; attitudes to eat healthy for the good taste of healthy foods, for overall health and for appearance; social norm on healthy eating or social support to eat healthy were examined in this regard. It was hypothesized that more frequent use of these apps would be associated with higher scores for the mentioned determinants, which in turn would be associated with lower BMI and healthier snacking and drinking habits.

## Methods

### Project

This research was conducted within the context of the Rewarding Healthy Food Choices Project [38], a multidisciplinary project that aims to investigate and improve the nutritional status of children and adolescents. The project combines rewarding paradigms with learning theory and typical behavior change techniques such as monitoring and goal setting, through novel methods such as serious games and mobile phone apps.

### Study Procedure and Participants

Data were collected using a pencil-and-paper survey from September 2013 to December 2013 in 14- to 16-year-old adolescents from 20 schools in Flanders, Belgium. A total of 1210 adolescents were sampled; the detailed sample size calculation and sampling procedure was already described elsewhere [1]. The adolescents completed the survey in the classroom in the presence of two researchers who provided clarification where necessary. Confidentiality and anonymity were assured by the researchers before, during, and after the completion of the survey. Passive consent was obtained from the parents or legal guardians of the sampled adolescents and the adolescents were informed that they could withdraw from the study at any time without explanation. The study protocol was approved by the Ethics Committee of the Ghent University Hospital.

### Measures

#### Demographics

Both sex and date of birth of the participants were recorded. Age was derived by subtracting the date of birth from the date the survey took place. The education type of each adolescent (general or technical or vocational) was obtained from the schools.

#### Snack and Beverage Intake

Snack and beverage intake were assessed using a food frequency questionnaire (FFQ), which is designed to measure snack and beverage intake of adolescents [39]. The FFQ probes for usual food intake with a reference period of 1 month and comprises two sections: beverages and snacks. The intake of beverages was evaluated over the whole day, as beverages such as soft drinks and fruit juice provide additional calories not only at snack times but also throughout the whole day [40]. The intake of snacks was evaluated in terms of all food items consumed outside (>30 min) of breakfast, lunch, and dinner [41].

Snacks and beverages were classified as either unhealthy or healthy using the UK Ofcom Nutrient Profiling model [42]. This model provides a score that represents the “unhealthiness” of a beverage or food product [42]. Following this scoring system, the snack and beverage items are sport drinks, energy drinks, soft drinks, sweetened milk drinks, crisps, other salty snacks, savory rolls (cheese or sausage) or pizza, other fried snacks, fries, hamburgers, cheese or meat cubes, sandwiches with sweet or savory spread, ice cream, popsicles, breakfast cereals, pudding, mousses, chocolate, candy bars, candy, dry cookies, other cookies, breakfast rolls, and pastries, which were all considered to be unhealthy. Items such as water, fruit or vegetable juice, coffee or tea, milk, milk substitutes, unsweetened yoghurt, sweetened yoghurt, dried fruit, fruit, raw vegetables, nuts and seeds, kebab and pasta cups were considered to be healthy.

For each FFQ category, the daily intake was calculated by multiplying the frequency of consumption with the quantity of consumption per week (g) divided by 7. These daily intakes were then summed to obtain the daily intake of healthy snacks (g), unhealthy snacks (g), unhealthy drinks (mL), and healthy drinks (mL). Subsequently, a healthy snack and a healthy beverage ratio were then calculated. These ratios were calculated as follows: gram healthy snacks or beverages/(gram healthy snacks or beverages + gram unhealthy snacks or beverages)×100. The higher this ratio, the more healthy the snack and beverage intake of the adolescents was.

#### Fitness and Nutrition App Use

Frequencies of fitness and nutrition app use were assessed with the questions: “How often do you use fitness apps on your mobile phone or tablet?” with examples Nike+Running and Fitness Pall and “How often do you use nutrition apps on your mobile phone or tablet?” with examples Weight Watchers and Calorie Counter. Response categories were (almost) *never*, *a few times a year*, *once a month*, *a few times per month*, *once every week*, *a few times per week*, and *(almost) daily*. The answer format was adapted from a previous study on the change in the frequency of media use among adolescents over time [43]. Response categories were rescaled to represent how many times such an app was used in 1 week. *Never* and *a few times a year* were set to 0, whereas other answer categories were given the following values: *once a month*=0.25 (reflected using the apps once every 4 weeks), *a few times per month*=0.5 (midpoint of the interval), *once every week*=1 (reflected using the apps one day a week), *a few times per week*=3.5 (midpoint of the interval), and *daily*=7 (reflected using the apps every day of the week).

#### Perceived Behavioral Control, Social Influence, and Attitudes

Perceived behavioral control to eat healthy, social norm of healthy eating, social support to eat healthy, and attitudes to eat healthy for the good taste of healthy foods, for overall health and for appearance were measured via 13 items taken from an existing valid and reliable healthy diet determinants questionnaire (Table 1) [35]. All items were evaluated using a 5-point Likert scale. For the constructs perceived behavioral control to eat healthy and attitude to eat healthy for overall health and for appearance, mean scores ranging from 1 to 5 were computed by averaging the scores of the items used to measure these constructs.

**Table 1:** Overview used constructs, items, and anchors of the healthy diet determinants questionnaire.

Constructs	Questions	Anchors	Cronbach alpha
Perceived behavioral control (1-5)	Suppose you want to eat healthy...How hard is it for you to eat healthy each day?	1=very hard and 5=not hard at all	.71
	How hard is it for you to eat a healthy diet at your home?		
	How hard is it for you to eat a healthy diet at your school?		
Peer social norm (1-5)	How healthy does your best friend eat?	1=very unhealthy and 5=very unhealthy	N/A^a^
Peer social support (1-5)	How often does your best friend encourage you to eat a healthy diet?	1=not at all and 5=very often	N/A
Attitude toward healthy eating for the good taste of healthy foods (1-5)	A reason or benefit for me to eat healthy is that I like the taste of healthy foods	1=completely disagree and 5=completely agree	N/A
Attitude toward healthy eating for overall health (1-5)	I think healthy eating is important for my overall health	1=completely disagree and 5=completely agree	.79
	A reason or benefit for me to eat healthy is that I feel better eating healthy	1=completely disagree and 5=completely agree	
	A reason or benefit for me to eat healthy is that I stay in good health		
Attitude toward healthy eating for appearance (1-5)	A reason or benefit for me to eat healthy is...that I lose weight	1=completely disagree and 5=completely agree	.79
	A reason or benefit for me to eat healthy is...that I can keep my weight as it is now and do not become overweight	1=completely disagree and 5=completely agree	
	A reason or benefit for me to eat healthy is...that other people admire me		
	A reason or benefit for me to eat healthy is...to have an attractive body		

^a^N/A: not applicable.

#### Height and Weight

Two trained research assistants measured body height and weight using a standardized protocol [44]. Adolescents were measured without shoes and were allowed to wear light clothing. Body height was measured with a SECA Leicester Portable Stadiometer with an accuracy of 1 mm. Weight was measured with a calibrated electronic scale SECA 861 with an accuracy of 100 g. Age and sex-specific body mass index z-scores (zBMI) were calculated using Flemish 2004 growth reference data [45]. According to the International Obesity Task Force cut-off points, adolescents were classified as either normal weight or overweight [46].

### Statistical Analyses

First, descriptive statistics of the sample were computed. Second, associations between the independent variables (fitness and/or nutrition app use frequency) and the dependent variables (zBMI, the healthy snack ratio, and the healthy beverage ratio) were assessed using multilevel linear regression analyses with a three-level structure (adolescents within classes within schools) to account for clustering of the data. Five consecutive models were tested. Model 0 was an intercept-only model without any level 1, level 2, or level 3 predictors; Model 1 was a covariates-only model (gender and education type). Models 2 and 3 evaluated the singular associations of fitness or nutrition app use frequency with the dependent variables by adding the fitness app use frequency or nutrition app use frequency to Model 1. Model 4 examined the independent influence of fitness and nutrition app use frequency by simultaneously adding both fitness app use frequency and nutrition app use frequency to Model 1. Model 5 explored the interplay between fitness and nutrition app use frequency by adding the fitness app use frequency × nutrition app use frequency interaction term to Model 4. When evidence of interaction was found in Model 5, a margins plot was computed to allow easier interpretation. Gender and education type (two dummies) were operationalized as categorical variables with 0=boys or general education. Frequencies of fitness and nutrition app use were treated as continuous predictors. As the intercept- and the covariates-only models (Models 0 and 1) were less relevant to test the postulated hypotheses, only Models 2-5 were presented.

Finally, to assess the mechanisms through which fitness and nutrition apps influence behavior, mediation analyses were executed for each app separately. Mediation of the associations between the independent (zBMI, healthy snack ratio, or healthy beverage ratio) and the dependent variables (fitness app use frequency or nutrition app use frequency) by the healthy diet determinants was explored with multiple mediation models. These models were fitted using multilevel structural equation modeling (MSEM; path analyses) with three levels for each of the app-outcome combinations, resulting in 6 models. Mediation was assessed following Preacher, Zyphur and Zhang [47,48] for the multilevel 1-1-1 model, using bootstrapped standard errors for the indirect effects (1000 replications). The coefficients shown in “Results” section, however, are the result of single level generalized structural equation modeling (GSEM) as the multilevel models did not provide substantial higher efficiency, based on Akaike information criterion (AIC), and computationally simpler models were thus preferred.

For both the multilevel regression models, as the multiple mediation models associations were controlled for gender and education type, continuous parameters were mean centered, outliers were removed, unstandardized coefficients and their standard errors were displayed, and associations with *P* value <.05 were considered statistically significant. For all models also the log-likelihoods and the log-likelihood tests compared with the null model (intercept only), together with the explained variances compared with the null model were computed. All analyses were conducted using Stata version 13 SE (StataCorp LP).

## Results

### Descriptives

Of the 1210 selected adolescents, 6% were absent or did not receive parental consent and 2.8% returned a questionnaire of unsatisfactory quality (more than 33% of the questions not completed or straight-lining responses). Only 73% (889/1104) of the adolescents who filled out the survey completed the questions on app use and were considered for the analyses. The mean age of these adolescents was 14.7 years 54.8% (481/878) were male, 18.1% (145/803) overweight or obese, 46.1% (410/889) enrolled in general, 34.7% (308/889) in technical, and 19.2% (171/889) in vocational education (see Table 2). Table 2 also shows the mean and standard deviations (SDs) for the dependent and independent variables. The mean zBMI was 0.28 (SD 1.02), the mean healthy snack ratio 37.16 (SD 25.39), and the mean healthy beverage ratio 72.76 (SD 24.79). Healthy snacks and beverages thus accounted for 37.2% and 72.8%, respectively, of the total snack or beverage intake in adolescents.

**Table 2:** Characteristics of the participants (n=889), zBMI, snack and beverage intake, perceived behavioral control, social influences, and attitudes.

Characteristics			% or mean (SD^a^)
**Demographics**			
	Overweight	18.1	
	Boys	4.8	
	General education	46.1	
	Technical education	34.7	
	Vocational education	19.2	
	Age	14.69 (0.81)	
**App use**			
	Use fitness apps	17.6%	
	Use nutrition apps	7.6%	
	Use both fitness and nutrition apps	1.7%	
	Frequency of use of fitness apps (0-7)	0.54 (1.47)	
	Frequency of use of nutrition apps (0-7)	0.16 (0.80)	
**zBMI^b^** **, healthy snack, and beverage ratio**			
	zBMI	0.28 (1.02)	
	Healthy snack ratio	37.16 (25.39)	
	Healthy beverage ratio	72.67 (24.79)	
**Healthy eating determinants**			
	Perceived behavioral control to eat healthy (1-5)	3.36 (0.82)	
	Attitude to eat healthy eating for the good taste of healthy foods (1-5)	3.70 (0.78)	
	Attitude to eat healthy for overall health (1-5)	3.70 (0.78)	
	Attitude to eat healthy for appearance (1-5)	3.03 (0.90)	
	Social norm to eat healthy (1-5)	3.12 (0.84)	
	Social support to eat healthy (1-5)	2.11 (0.95)	

^a^SD: standard deviation.

^b^zBMI: body mass index z-scores.

A total of 27.6% (245/889) of the adolescents used fitness and nutrition apps, most of them used fitness apps (17.6%, 167/889). A smaller group used both fitness and nutrition apps (7.6%, 63/889) and merely 3% (15/889) used only nutrition apps. The mean frequency of use is less than once a month for nutrition apps and between a few times per month and every week for fitness apps.

### Multilevel Associations

#### zBMI

**Figure 1:**
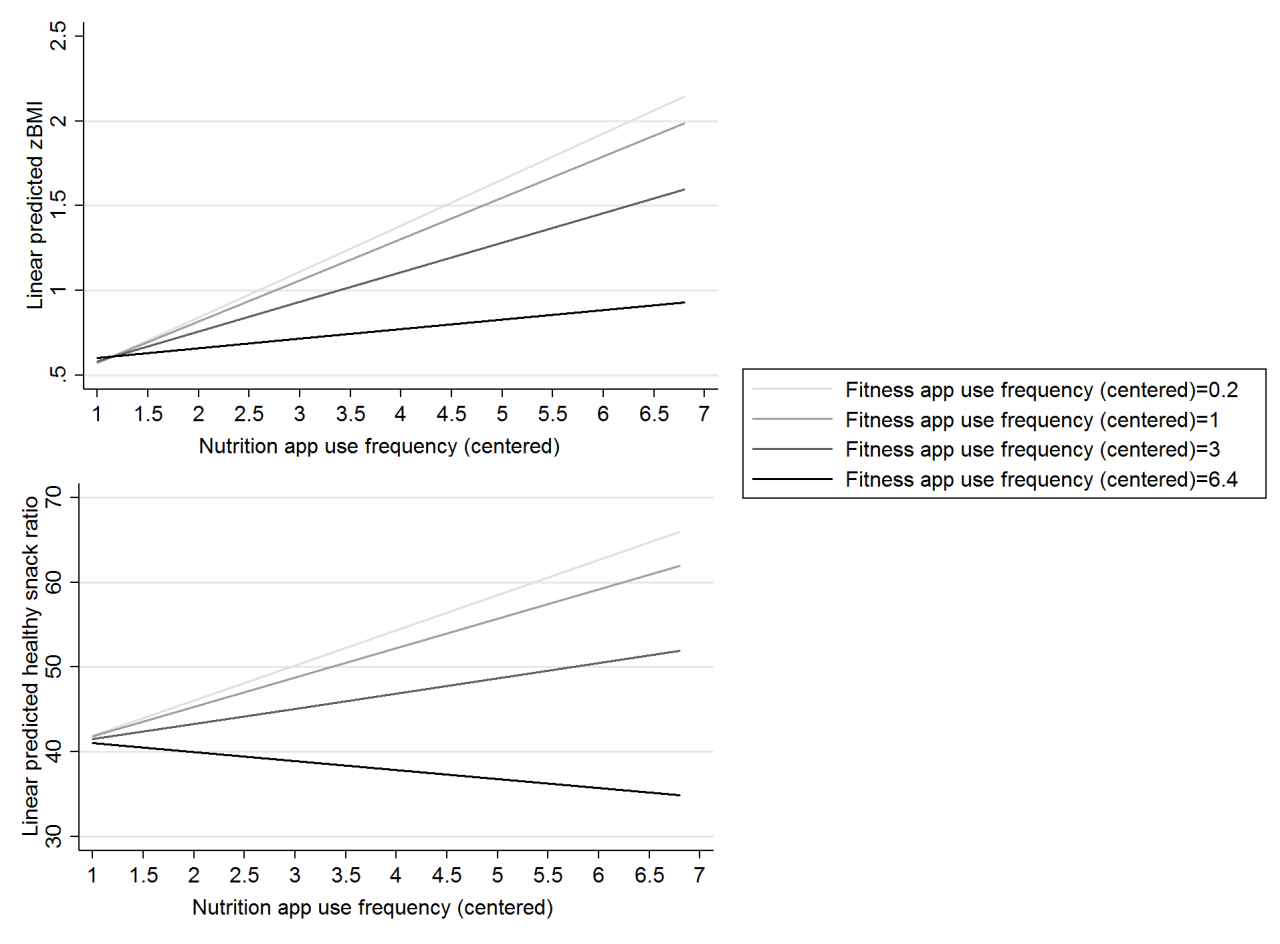
Margins plot zBMI and healthy snack ratio. Analyses controlled for sex and education type.

Both fitness and nutrition app use frequency were singularly associated with zBMI (see Models 2 and 3 in Multimedia Appendix 1). The more frequent adolescents used fitness apps (*b*=0.07 (0.02), *P*=.001) or nutrition apps (*b*=0.18 (0.03), *P*<.001), the higher the zBMI of the adolescents was. However, when both fitness and nutrition app use frequency were considered simultaneously (Model 4 in Multimedia Appendix 1), only nutrition app use frequency was independently and directly associated with zBMI (*b*=0.16 (0.04), *P*<.001). In addition, a significant interaction between fitness app use frequency and nutrition app use frequency (*b*=−0.03 (0.02), *P*=.04) in relation to zBMI was found (Model 5 Multimedia Appendix 1) in a way that when fitness apps were more frequently used, the association between nutrition app use frequency and zBMI decreased (see margins plot in Figure 1). Model 5 had the lowest log-likelihood and was thus the best fitting model. Together with the covariates, fitness and nutrition app use frequencies explained 7% of the variation in zBMI.

#### Healthy Snack Ratio

No significant singular or independent associations between the frequencies of use of fitness or nutrition apps and the healthy snack ratio could be observed (see Models 2-4 in Multimedia Appendix 1). However, there was an interaction of fitness and nutrition app use frequency (*b*=−0.84 (0.37), *P*=.03) with the healthy snack ratio (Model 5 in Multimedia Appendix 1). More specifically, the association between the nutrition app use frequency and the healthy snack ratio was positive at low frequencies of use of fitness apps. At higher frequencies, the association between nutrition app use frequency and the healthy snack ratio was negative (Figure 1). Here also, Model 5 was the best fitting model. The covariates together with the frequencies of use of both fitness and nutrition apps explained 9% of the variation in the healthy snack ratio.

#### Healthy Beverage Ratio

In contrast to fitness app use frequency, nutrition app use frequency was significantly associated with the healthy beverage ratio (see Models 2-4 in Multimedia Appendix 1). Adolescents who used nutrition apps more often had a higher healthy beverage ratio (*b*=2.96 (1.11), *P*=.008). Also, no significant interaction of fitness app use frequency and nutrition app use frequency was observed (see Model 5 in Multimedia Appendix 1). Model 5 again had the lowest log-likelihood and thus provided the best fit. Together with the covariates, fitness and nutrition app use frequencies explained 5% of the variation in the healthy beverage ratio.

### Multiple Mediation

**Figure 2:**
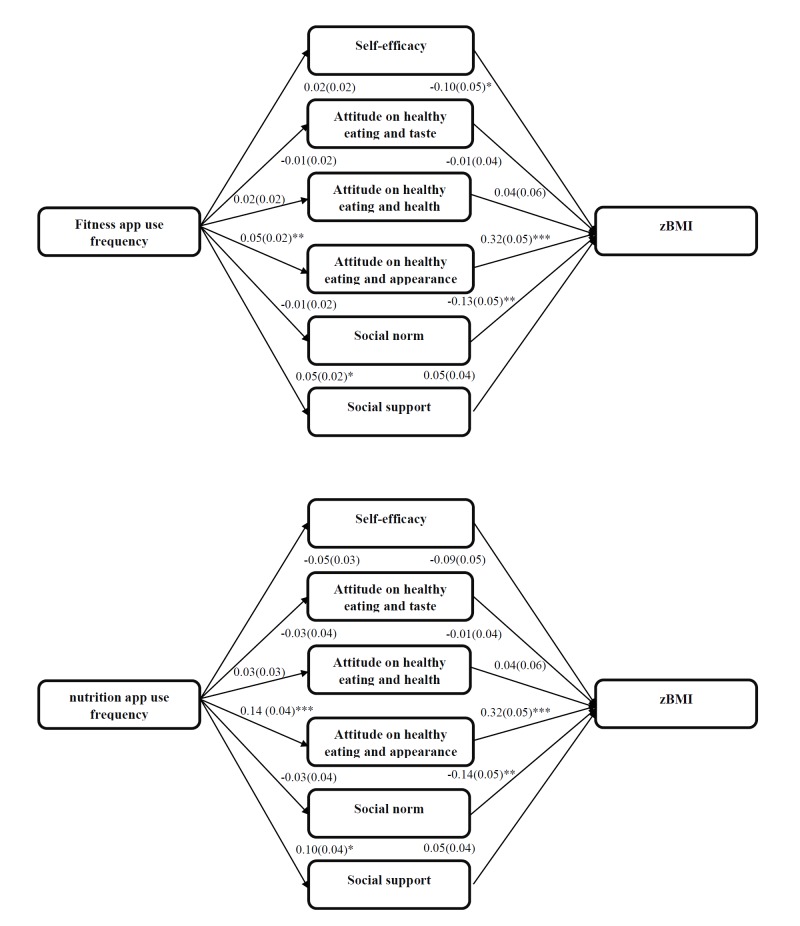
Multiple mediation zBMI. Analyses controlled for sex and education type; * P<.05, ** P <.01, *** P <.001.

**Figure 3:**
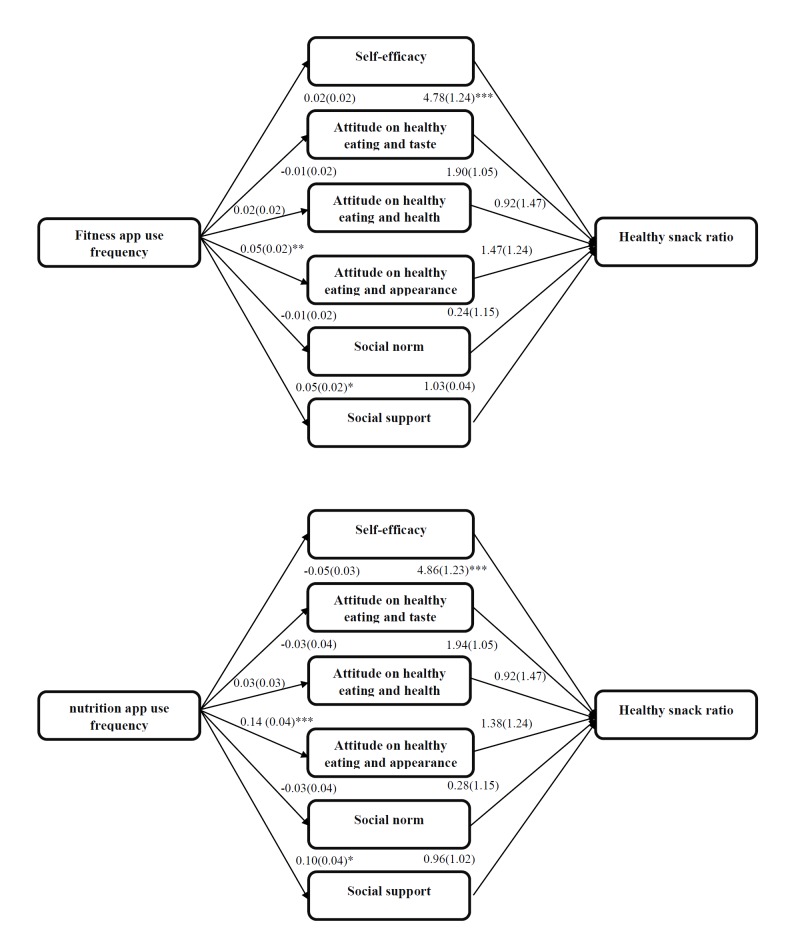
Multiple mediation healthy snack ratio. Analyses controlled for sex and education type; * P <.05, ** P <.01, *** P <.001.

**Figure 4:**
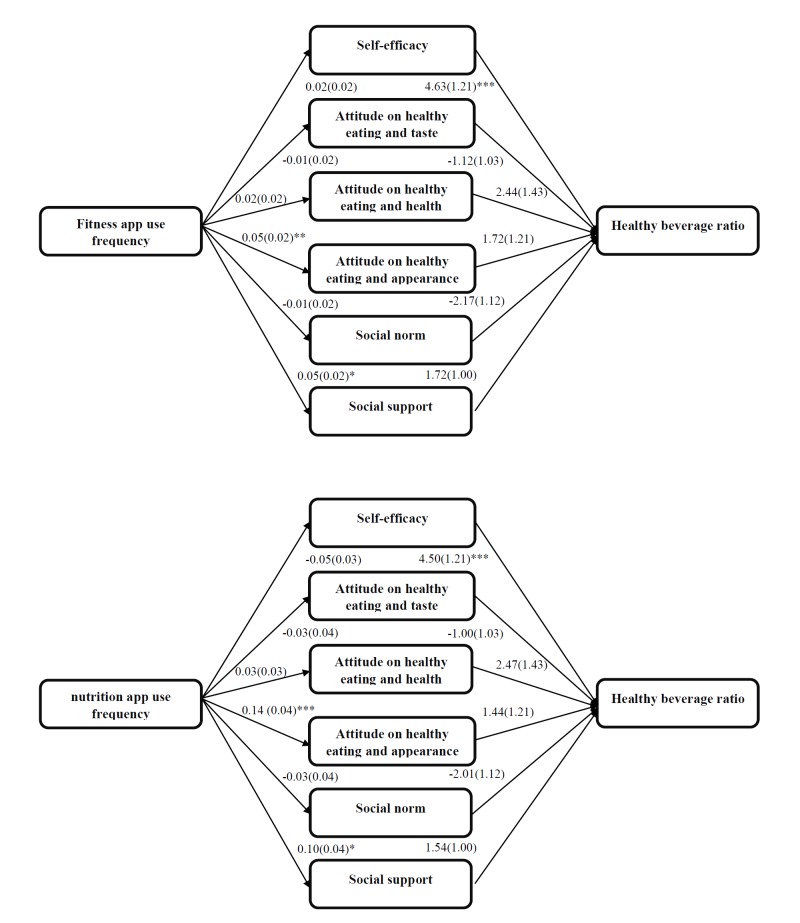
Multiple mediation healthy beverage ratio. Analyses controlled for sex and education type; * P <.05, ** P <.01, *** P <.001.

The multiple mediation analyses indicated that both the fitness and nutrition app use frequencies were positively associated with social support to eat healthy (fitness apps *b*=0.05 [0.02], *P*=.03; nutrition apps *b*=0.10 [0.04], *P*=.01) and attitude to eat healthy for appearance (fitness apps *b*=0.05 [0.02], *P*=.008; nutrition apps *b*=0.14 [0.04], *P*<.001) (see Figures 2-4, top). The higher the app use frequency, the more positive the attitude to eat healthy for appearance and the felt social support. However, only attitude to eat healthy for appearance was found to be a mediator. The attitude to eat healthy for appearance mediated both the fitness app use frequency-zBMI relation (*a* × *b*=0.02 [0.01], *P*=.02) and the nutrition app use frequency-zBMI relation (*a* × *b*=0.04 [0.01], *P*=.001) (see Figure 3 and Tables 3 and 4). The higher the frequencies of use of fitness or nutrition apps, the more positive the attitude to eat healthy for appearance and the higher the zBMI. The associations between the frequencies of use of nutrition or fitness apps and the healthy snack ratio or the healthy beverage ratio were not mediated by any of the proposed mediators. The multiple mediation models explained, respectively, 9%, 11%, and 16% of the variance in zBMI, the healthy snack ratio, and the healthy beverage ratio (see Figures 2-4).

**Table 3:** Indirect effects multiple mediation with fitness app use frequency.

Mediator		Indirect effect (*a* × *b*)	Bootstrapped SE^a^	*z*	*P*	Normal-based 95% CI
**zBMI^b^**						
	Perceived behavioral control to eat healthy	−0.00	0.00	−0.89	.37	−0.01 to 0.00
	Attitude to eat healthy for the good taste of healthy foods	0.00	0.00	0.03	.98	−0.00 to 0.00
	Attitude to eat healthy for overall health	0.00	0.00	0.28	.78	−0.00 to 0.00
	Attitude to eat healthy for appearance	0.02	0.01	2.28	.02	0.00-0.03
	Social norm to eat healthy	0.00	0.00	0.28	.78	−0.01 to 0.01
	Social support to eat healthy	0.00	0.00	0.99	.32	−0.00 to 0.01
	Total indirect effect	0.02	0.01	2.00	.046	0.00 to 0.03
**Healthy snack ratio**						
	Perceived behavioral control to eat healthy	0.11	0.11	1.01	.31	−0.10 to 0.32
	Attitude to eat healthy for the good taste of healthy foods	−0.01	0.05	−0.19	.85	−0.11 to 0.09
	Attitude to eat healthy for overall health	0.01	0.04	0.32	.75	−0.07 to 0.10
	Attitude to eat healthy for appearance	0.08	0.07	1.07	.29	−0.06 to 0.22
	Social norm to eat healthy	−0.00	0.03	−0.06	.96	−0.05 to 0.05
	Social support to eat healthy	0.05	0.06	0.81	.42	−0.07 to 0.17
	Total indirect effect	0.24	0.18	1.30	.20	−0.12 to 0.60
**Healthy beverage ratio**						
	Perceived behavioral control to eat healthy	0.10	0.10	1.04	.30	−0.09 to 0.30
	Attitude to eat healthy for the good taste of healthy foods	0.01	0.04	0.15	.88	−0.07 to 0.08
	Attitude to eat healthy for overall health	0.04	0.06	0.62	.54	−0.08 to 0.15
	Attitude to eat healthy for appearance	0.09	0.08	1.11	.26	−0.07 to 0.27
	Social norm to eat healthy	0.01	0.05	0.24	.81	−0.09 to 0.12
	Social support to eat healthy	0.08	0.07	1.24	.22	−0.05 to 0.21
	Total indirect effect	0.33	0.17	1.97	.049	0.00 to 0.66

^a^SE: standard error.

^b^zBMI: body mass index z-scores.

**Table 4:** Indirect effects multiple mediation with nutrition app use frequency.

Mediator		Indirect effect (*a* × *b*)	Bootstrapped SE^a^	*z*	*P*	Normal-based 95% CI
**zBMI^b^**						
	Perceived behavioral control to eat healthy	0.00	0.01	0.87	.39	−0.01 to 0.02
	Attitude to eat healthy for the good taste of healthy foods	−0.00	0.00	0.05	.69	−0.00 to 0.00
	Attitude to eat healthy for overall health	0.00	0.00	0.29	.77	−0.01 to 0.01
	Attitude to eat healthy for appearance	0.04	0.01	3.23	.001	0.02 to 0.07
	Social norm to eat healthy	0.00	0.00	0.99	.32	−0.00 to 0.01
	Social support to eat healthy	0.01	0.01	0.97	.33	−0.01 to 0.02
	Total indirect effect	0.06	0.02	3.75	<.001	0.03 to 0.09
**Healthy snack ratio**						
	Perceived behavioral control to eat healthy	−0.26	0.22	−1.17	.24	−0.70 to 0.17
	Attitude to eat healthy for the good taste of healthy foods	−0.05	0.12	−0.42	.68	−0.29 to 0.19
	Attitude to eat healthy for overall health	0.03	0.10	0.29	.77	−0.16 to 0.22
	Attitude to eat healthy for appearance	0.19	0.17	1.12	.26	−0.14 to 0.52
	Social norm to eat healthy	−0.01	0.05	−0.18	.85	−0.10 to 0.08
	Social support to eat healthy	0.10	0.13	0.73	.46	−0.16 to 0.36
	Total indirect effect	−0.01	0.38	0.02	.99	−0.30 to 0.87
**Healthy beverage ratio**						
	Perceived behavioral control to eat healthy	−0.24	0.20	−1.23	.22	−0.63 to 0.15
	Attitude to eat healthy for the good taste of healthy foods	0.02	0.08	0.32	.75	−0.14 to 0.19
	Attitude to eat healthy for overall health	0.08	0.14	0.56	.58	−0.19 to 0.34
	Attitude to eat healthy for appearance	0.20	0.20	1.01	.31	−0.19 to 0.58
	Social norm to eat healthy	0.06	0.07	0.91	.37	−0.08 to 0.21
	Social support to eat healthy	0.15	0.14	1.12	.26	−0.12 to 0.43
	Total indirect effect	0.28	0.35	0.79	.43	−0.41 to 0.96

^a^SE: standard error.

^b^zBMI: body mass index z-scores.

## Discussion

### Principal Findings

This study is one of the first to investigate associations, both independent and interactive, between commercial fitness and nutrition app use frequencies and adolescents’ snacking and drinking behaviors and BMI. A more frequent use of fitness and nutrition apps was associated with healthier drinking habits and a lower BMI. This study also assessed which determinants mediated the relations between the use of fitness or nutrition apps and BMI, healthy snacking, or healthy drinking habits. Only attitude to eat healthy for appearance was found to be a mediator of the relations between the frequency of use of fitness or nutrition apps and zBMI.

First, a total of 28% of the Flemish adolescents in our sample reported to use fitness or nutrition apps in 2013. The mean frequency of using fitness apps was between a few times per month and every week and less than once a month for nutrition apps. A study conducted in the United States in 2015 found that around 58% of the US adults had downloaded a health app and that 65% of these also used this app daily [13]. Apart from the country and the timing of the survey, the discrepancy between our results and the 2015 US survey could also be attributed to the surveyed population group. Possibly, adolescents use health apps less frequently when compared with adults. More in-depth research on the motives of adolescents to use health apps will be needed in the future to understand why adolescents might be less inclined to use such app when compared with adults.

Second, a higher use of nutrition apps was independently associated with a higher zBMI. This was unexpected, possibly adolescents using nutrition apps in this study were trying to lose weight. Results confirmed that nutrition app users were indeed more likely to be overweight (36% overweight) in comparison to adolescents who do not use these apps (16% overweight). Using nutrition apps could thus be part of interventions to lose weight. A desire to lose weight was one of the most frequent reasons to download a nutrition app in the United States [13]. Also, the more adolescents used nutrition apps, the more healthy their beverage intake was. No other studies are available to compare these findings with. No significant independent association between fitness apps use frequency and the healthy beverage ratio was found, nor were there significant independent associations observed between fitness or nutrition apps use frequency and the healthy snack ratio. The use of commercial fitness and nutrition apps was thus only weakly associated with healthier snacking and drinking habits in adolescents. This limited influence might be a consequence of their often limited theoretical ground. Our results support the conclusions from reviews and content analyses, which indicated that commercial fitness and nutrition apps tend to lack a thorough theoretical base and therefore might not be effective in promoting good health [14,15,24,29,31].

Third, evidence of an interaction between the frequencies of use of fitness and nutrition apps was found for zBMI and the healthy snack ratio, but not for the healthy beverage ratio. Frequently using both fitness and nutrition apps was associated not only with a lower BMI, but also with a lower healthy snack ratio. The latter finding was unexpected; this could however be a consequence of the perceived higher energy-needs of those adolescents who frequently use fitness apps. These fitness app users might consume more energy-bars that contain large amounts of sugar and/or fat. More research will be needed to further confirm our findings and to explore the existence of such interactive influences for other health behaviors as well. Research on adolescents’ motives for using and downloading fitness and/or nutrition apps would also be helpful for a better understanding and explanation of these interactions. Fourth, higher frequencies of fitness and nutrition apps use were associated with a more positive attitude to eat healthy for appearance, which was in turn associated with a higher zBMI. Adolescents with a higher BMI might thus use fitness or nutrition apps to look good or to lose weight. Future research could investigate whether these adolescents show dieting or restrained eating practices and if these practices can (partially) explain the association between nutrition or fitness app use and a higher zBMI. No evidence of mediation was found for the associations between the fitness or nutrition apps use frequency and the healthy snack or beverage ratio. In general, little evidence of mediation was found; current commercially available fitness and nutrition apps seem to influence only a few key determinants of eating behaviors. The apps might hence not incorporate the corresponding behavior change techniques or use these techniques in an effective way. Our findings thereby confirm those from several reviews and content analyses [14,15,24,29,31] that report beneficial influence of commercial fitness and nutrition apps on health is limited by their lack of (effective) behavior change techniques. Apps aimed to change behavior should thus focus more on targeting the key determinants identified in the literature and incorporate the corresponding behavior change techniques in an effective way [12,29].

### Strengths and Limitations

The strengths of this study were the use of a representative sample, the objective measurements of height and weight, the use of multilevel regression models and structural equation modeling (SEM) to research the associations and mediations. This study also has some limitations. First, this study considered the use of general fitness and nutrition apps with a 12+ rating. To date, only a few available health apps are specifically developed for adolescents, and adolescents will thus use general health apps. No assessment of the developmental appropriateness of such apps was made. However, such an analysis is warranted given that adolescents have other needs than adults, for example, simpler interfaces and different app features based on differentially identified behavior change techniques compared with adults [12,49,50]. Unfortunately, at present, no coding system and legalities related to age appropriateness of apps for children and adolescents exist within the regulatory framework of the EU [51]. Second, given the cross-sectional nature of this study design, no statements about the causality of the associations found could be made. Experimental research is therefore needed to further examine how nutrition and/or fitness app use influences BMI and eating behaviors or vice versa.. Third, all collected data except the anthropometrics were self-reported and therefore subject to social-desirability bias. Fourth, physical activity and total energy intake were not assessed as this would have increased the participant burden considerably. The survey was already quite lengthy (75 min), which could have increased the chance of poor quality answers at the end of the survey [52]. Three versions of the questionnaire, in which the question were placed in another order, were prepared and administered randomly (except for the demographics, these were always presented first).

### Conclusions

A more frequent use of a commercial nutrition app was independently associated with healthier drinking habits in adolescents, but with a higher zBMI. The interactive influence of frequently using both fitness and nutrition apps, on the other hand, was associated with a lower zBMI and less healthy snacking habits. In addition, no evidence of mediation by key determinants was found. Fitness and nutrition apps show some association with healthier eating behaviors in adolescents, but their potential for health promotion could probably be enhanced by incorporating more (effective) behavior change techniques.

### Implications and Future Research

The present study was a first attempt to map adolescents’ use of commercial health apps and to investigate the relation of using these apps with adolescents’ health status in terms of snacking and drinking habits and BMI. Further research is needed to more fully comprehend adolescents’ motives for using and downloading such apps. Future research should also continue to explore adolescents’ use of commercial nutrition and fitness to determine the possible usefulness of the current fitness and nutrition apps for health promotion among adolescents.

Better understanding of commercial fitness and nutrition app use in adolescents can also guide efforts to develop effective smartphone interventions for healthy lifestyles. In addition to further researching the mechanisms of actions, future studies could also explore what features of commercial apps are deemed effective and liked by adolescents. Evidence from adults [10,13,26,53] in this regard cannot be extended to adolescents, as adolescents have different preferences and need different behavior change techniques than adults [12].

The demand for apps that promote healthy habits is high and these apps are assumed to have a substantial potential for health promotion initiatives. At this point, however, few effective theory-based apps are available on the app market, especially for adolescents. Public health professionals and app developers should collaborate to design more theory-based apps to be used in health promotion and fulfill the needs of the population [29]. Future research should thus focus on developing such apps, by translating and incorporating the already identified effective behavior change techniques into mobile apps and conducting experimental trials to investigate their effectiveness on the behavior of interest and its related determinants [29,31].

## Appendix

Multimedia Appendix 1 Associations between zBMI, healthy snack ratio, healthy beverage ratio, and fitness and nutrition app use frequency.

## Abbreviations

AIC: Akaike information criterion
BMI: body mass index
FFQ: food frequency questionnaire
GSEM: generalized structural equation modeling
MSEM: multilevel structural equation modeling
zBMI: body mass index z-score
