# Supplementary material for: Use of Fitness and Nutrition Apps: Associations With Body Mass Index, Snacking, and Drinking Habits in Adolescents
Source: JMIR Mhealth Uhealth. 2017 Apr 25;5(4):e58. doi: 10.2196/mhealth.6005 (PMC5424128; doi:10.2196/mhealth.6005)
Supplement: Multimedia Appendix 1 [file mhealth_v5i4e58_app1.pdf]

Dependent variables	Independent variables	Model 2		Model 3		Model 4		Model 5	
		b (SE[a])	*P*	*b* (SE[a])	*P*	*b* (SE[a])	*P*	*b* (SE[a])	*P*
**zBMI[b]**									
	Frequency of fitness app use	0.06 (0.02)	.009			0.03 (0.03)	.25	0.04 (0.03)	.15
	Frequency of nutrition app use			0.16 (0.05)	<.001	0.13 (0.05)	.008	0.28 (0.09)	.001
	Frequency of nutrition app use×frequency of fitness app use							−0.03 (0.02)	.04
	Log-likelihood	−1139.82		−1136.98		−1136.33		−1134.14	
	2 Δ Log-likelihood (Δdf)	48.52 (3)	<.001	54.2 (3)	<.001	55.50 (4)	<.001	59.88 (5)	<.001
	Explained variance	0,04		0,04		0,04		0,05	
**Healthy snack ratio**									
	Frequency of fitness app use	0.72 (0.56)	.20			0.47 (0.62)	.45	0.70 (0.62)	.26
	Frequency of nutrition app use			1.45 (1.03)	.15	1.09 (1.14)	.34	4.31 (1.83)	.02
	Frequency of nutrition app use× frequency of fitness app							−0.84 (0.37)	.03

	use								
	Log-likelihood	−3983.32		−3983.14		−3982.86		−3980.34	
	2 Δ Log-likelihood (Δdf)	172.16 (3)	<.001	172.52 (3)	<.001	173.08 (4)	<.001	178.12 (5)	<.001
	Explained variance	0,08		0,08		0,08		0,09	
**Healthy beverage ratio**									
	Frequency of fitness app use	−0.24 (0.55)	.66			−0.92 (0.61)	.13	−0.83 (0.62)	.52
	Frequency of nutrition app use			2.26 (1.01)	.03	2.96 (1.11)	.008	4.12 (1.79)	.02
	Frequency of nutrition app use× frequency of fitness app use							−0.30 (0.37)	.41
	Log-likelihood	−4026.81		−4024.42		−4023.30		−4022.96	
	2 Δ Log-likelihood (Δdf)	123.26 (3)	<.001	128.04 (3)	<.001	130.28 (4)	<.001	130.96 (5)	<.001
	Explained variance	0,04		0,05		0,05		0,05	

[a] SE: standard error.
[b] zBMI: body mass index z-scores.
